# Excessively Low Insulin Resistance May Increase the Risk of All-Cause Mortality Among Community-Dwelling Individuals Without Diabetes

**DOI:** 10.7759/cureus.81773

**Published:** 2025-04-05

**Authors:** Ryuichi Kawamoto, Asuka Kikuchi, Daisuke Ninomiya, Teru Kumagi, Masanori Abe

**Affiliations:** 1 Department of Community Medicine, Ehime University Graduate School of Medicine, Toon, JPN

**Keywords:** all-cause mortality, body mass index, insulin resistance, interaction, japanese population

## Abstract

Background

Epidemiological evidence has indicated that insulin resistance (IR), as measured by a homeostatic model assessment for IR (HOMA-IR), is strongly correlated with body mass index (BMI). However, there is a paucity of studies assessing the complex interaction between BMI and HOMA-IR with respect to all-cause mortality, particularly among Asian individuals without diabetes.

Materials and methods

The research centered on individuals diagnosed without diabetes, comprising 881 men with a mean age of 62 years (± standard deviation (SD): 14) and 1,159 women with a mean age of 64 years (± 11). The participants were drawn from the Nomura cohort study, consisting of two cohorts: one initiated in 2002 and the other in 2014. To assess the risk of all-cause mortality up to the end of the follow-up period, we applied a Cox proportional hazards model, adjusting for a range of covariates to calculate the hazard ratios (HRs).

Results

Participants were followed for a median duration of 7,691 days (interquartile range: 4,235-7,761 days). Over the course of the follow-up period, a total of 672 deaths were documented, comprising 338 deaths among men and 334 among women. The interaction between BMI and HOMA-IR (HR: 1.05; 95% confidence interval (CI): 1.02-1.09) was significantly associated with all-cause mortality, along with gender, age, BMI, history of cardiovascular disease, hyperuricemia, and HOMA-IR. Moreover, the HRs for all-cause mortality were examined for each BMI group by dividing the HOMA-IR by one SD. In the BMI < 22.0 kg/m² group, using the third HOMA-IR as the reference, significant HR (J curve) increases were observed in the first, second, and fourth HOMA-IR. In the BMI ≥ 22.0 kg/m² group, using the first HOMA-IR as the reference, a significant increase in HR was observed only in the fourth HOMA-IR. An interaction between BMI and HOMA-IR was identified for all-cause mortality (p = 0.005).

Conclusions

BMI confounds the association between IR, as measured by HOMA-IR, and the risk of all-cause mortality among Japanese individuals.

## Introduction

Insulin resistance (IR) is a key characteristic of metabolic syndrome, indicating a reduced effectiveness of insulin in facilitating glucose uptake by peripheral cells such as adipose tissue, skeletal muscle, liver, and endothelial tissue [1]. IR is recognized as a risk factor for both microvascular and macrovascular complications [2] and is associated with the onset of hyperglycemia, increased blood pressure [3], nonalcoholic fatty liver disease [4], atherogenic dyslipidemia [5], and a variety of other malignancies [6]. Meta-analyses have indicated that IR is independently linked to an increased risk of all-cause mortality among nondiabetic adults [7].

A validated alternative metric for evaluating IR is the homeostatic model assessment of IR (HOMA-IR), derived from fasting blood glucose (FBG) and insulin levels [8]. Song et al. [9] demonstrated that HOMA-IR was an independent and consistent predictor of diabetes risk in a diverse cohort of 82,069 postmenopausal women in the United States. Previous cross-sectional studies have shown that elevated HOMA-IR is associated with a higher prevalence of impaired glucose tolerance (IGT) and type 2 diabetes [10,11]. Additionally, several prospective studies have emphasized the role of HOMA-IR in predicting the future risk of type 2 diabetes and/or IGT across diverse populations [12]. HOMA-IR tests have been developed and accepted for epidemiological or clinical studies [13].

Obesity is widely recognized as a significant risk factor for IR, especially when it involves ectopic fat accumulation rather than subcutaneous fat [14]. Furthermore, several non-obesity-related factors also play a crucial role in the development of IR. These include race, gender, physical activity, and genetic predispositions. Furthermore, other unknown causes are likely contributing to IR [15].

However, few studies investigate the long-term effects of IR, particularly at low levels in individuals without diabetes. In this study, we investigated the distribution of HOMA-IR divided by body mass index (BMI) and how this metric relates to all-cause mortality in participants. Additionally, we analyzed cohort data to evaluate age as a potential risk factor.

## Materials and methods

Study design and participants

**Figure 1:**
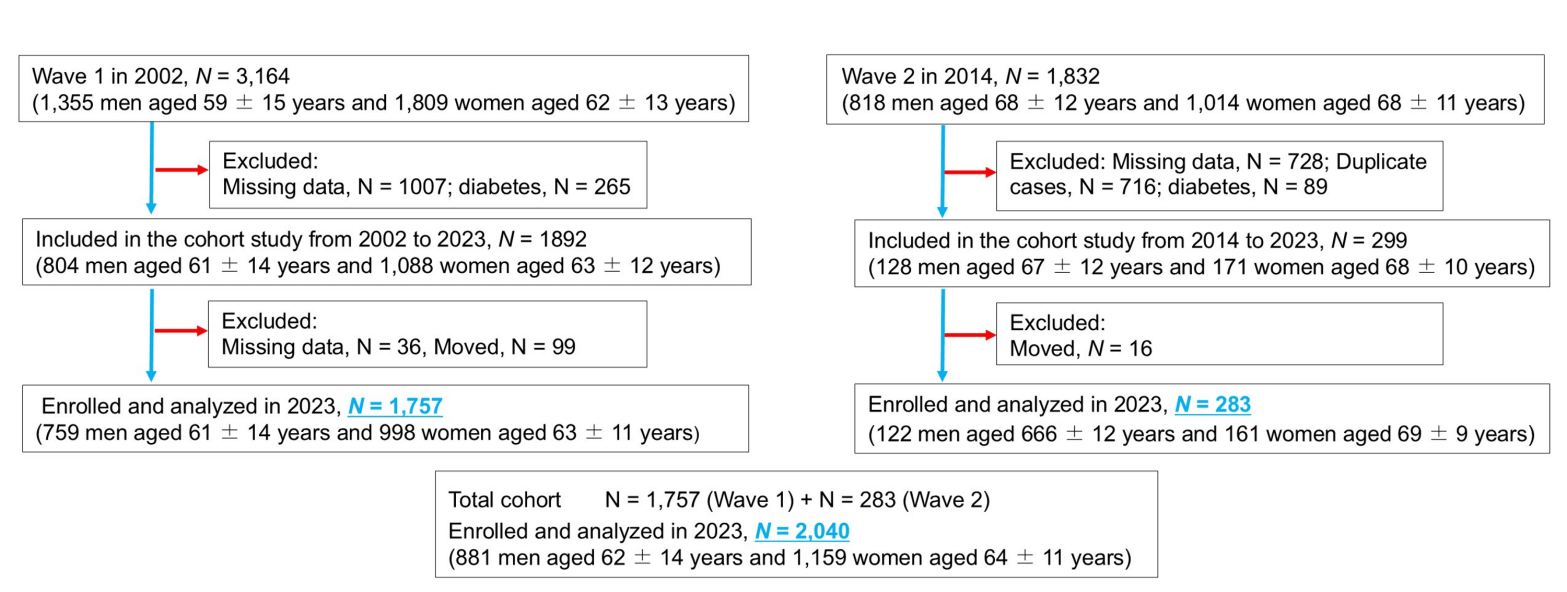
Flowchart of participants

The study included individuals predominantly residing in rural areas of Ehime Prefecture who participated in community-based annual health check-ups. The target population consisted of individuals without diabetes, with FBG levels below 126 mg/dL and not receiving antidiabetic treatment. The initial cohort (established in 2002) comprised 3,164 participants, while the second cohort (established in 2014) included 1,832 participants, ages 23 to 89 years. Among them, 1,892 participants from the first cohort and 299 from the second cohort completed baseline physical examinations and were subsequently included in the follow-up study. Data from 2,040 participants across both cohorts were analyzed (Figure 1). A self-administered questionnaire captured information on lifestyle habits, medical history, current health status, and medication use. Participant inclusion and exclusion were guided by flowcharts from previous studies [16]. The first cohort underwent follow-up surveys over 21 years, while the second cohort was followed for 10 years. Participant survival status was confirmed using Japan’s Basic Resident Register (December 2023).

Study approval

The study was approved by the Institutional Review Board of Ehime University Hospital (approval number 1903018). Written informed consent was obtained from all participants after they were provided with comprehensive explanations of the study's objectives and procedures.

Assessment of risk factors

The study collected data on participants' weight and height, with BMI determined by dividing weight (kg) by height squared (m²). Smoking history was evaluated in terms of pack-years, determined by multiplying the total duration of smoking (in years) by the average number of packs smoked per day. This measure grouped participants into four categories: non-smokers, former smokers, light smokers (fewer than 20 pack-years), and heavy smokers (20 or more pack-years). Alcohol consumption was measured in sake units, where one unit is equivalent to 22.9 g of ethanol. Participants were categorized into four drinking groups: abstainers, occasional drinkers (less than one unit per day), light daily drinkers (one to two units per day), and heavy daily drinkers (two to three units per day). Notably, none of the participants reported consuming more than three units per day.

Systolic blood pressure (SBP) and diastolic blood pressure (DBP) were measured twice on the right upper arm of participants in a seated position, following a minimum rest period of five minutes. Measurements were taken using an appropriately sized cuff and an automatic oscillometric blood pressure recorder (BP-103i; Colin, Aichi, Japan), and the average of the two readings was used.

Blood tests were performed to assess various parameters, including triglycerides (TG), high-density lipoprotein cholesterol (HDL-C), low-density lipoprotein cholesterol (LDL-C), FBG, creatinine (Cr), serum uric acid (SUA), and immunoreactive insulin (IRI). HOMA-IR was calculated using the following formula: FBG (mg/dL) × IRI (μU/mL)/405 [17]. The estimated glomerular filtration rate (eGFR) was determined using the Chronic Kidney Disease Epidemiology Collaboration (CKD-EPI) equation, adjusted with coefficients specific to the Japanese population. For males with serum Cr levels ≤ 0.9 mg/dL, the formula was 141 × (Cr/0.9) ^-0.411^ × 0.993 ^age^ × 0.813, while for Cr levels > 0.9 mg/dL, it was 141 × (Cr/0.9) ^-1.209^ × 0.993 ^age^ × 0.813. Similarly, for females with Cr levels ≤ 0.7 mg/dL, the formula used was 144 × (Cr/0.7) ^-0.329^ × 0.993 ^age^ × 0.813, and for Cr levels > 0.7 mg/dL, it was 144 × (Cr/0.7) ^-1.209^ × 0.993 ^age^ × 0.813 [18].

Participants were classified as hypertensive if their SBP was ≥ 140 mmHg, their DBP was ≥ 90 mmHg, or if they were receiving antihypertensive treatment. The classification was based on predefined criteria. Hypertriglyceridemia was defined as TG levels ≥ 150 mg/dL, while hypo-HDL cholesterolemia was diagnosed in participants with HDL-C levels ≤ 40 mg/dL. Hyperlipidemia was determined by LDL-C levels ≥ 140 mg/dL or lipid-lowering medications. Hyperuricemia was identified in individuals with SUA levels ≥ 7.0 mg/dL, including those on SUA-lowering therapy. CKD was an eGFR < 60 mL/min/1.73 m². Lastly, participants were categorized as having cardiovascular disease (CVD) if they had a history of conditions such as ischemic heart disease, ischemic stroke, or peripheral vascular disease.

Statistical analysis

The data were subjected to statistical analysis using SPSS Statistics version 27.0 (IBM Corp. Released 2020. IBM SPSS Statistics for Windows, Version 27.0. Armonk, NY: IBM Corp). If the data were normally distributed, we used the mean and standard deviation (SD) to express continuous variables. If the data were not normally distributed, such as in TG, FBG, IRI, and HOMA-IR, we used the median (interquartile range). Log-transformed values were used for parameters with non-normal distributions in all analyses. Participants were divided into two groups based on a BMI of either above or below 22.0 kg/m^2^, the highest life expectancy value of Japanese individuals [19], and into four groups by one SD of HOMA-IR. Chi-square tests were applied to compare categorical variables, while the Student’s t-test was used for continuous variables with a normal distribution. Baseline characteristics were individually assessed using the Cox proportional hazards model to identify significant confounders, which were subsequently incorporated as covariates. Multivariable analysis was then performed using the forced-entry method within the Cox proportional hazards model, with age as the primary time variable. Interaction analysis was conducted to examine the influence of the variable of interest, with adjustments made for all significant confounders (excluding the variable under evaluation). Statistical significance was determined using two-tailed p-values with a threshold set at p < 0.05.

## Results

Baseline characteristics of subjects categorized by BMI

The sample consisted of 2,040 participants, with males representing 43.2% of the total population. The average age for males was 62 ± 14 years, while for females, it was 64 ± 11 years. The median follow-up period was 7,691 days (interquartile range: 4,235-7,761 days). During this period, 672 deaths occurred, which represented 32.9% of all participants. Of these, 338 were males (38.4% of all males) and 334 were females (28.8% of all females). Table 1 presents the baseline characteristics of participants, categorized by baseline BMI. In the group with a BMI of 22.0 kg/m² or higher, the proportion of males and the prevalence of hypertension, hypertriglyceridemia, hypo-HDL cholesterolemia, hyperlipidemia, CKD, hyperuricemia, and HOMA-IR were all significantly higher compared to the group with a BMI of less than 22.0 kg/m². No significant differences were observed between the two groups regarding age, smoking status, drinking habits, or history of CVD.

**Table 1:** Baseline characteristics of subjects categorized by BMI Data are presented as mean ± SD. Data for TG, HOMA-IR, and FBG were skewed and are thus presented as median (interquartile range) values and log-transformed for analysis. * Chi-square tests were used to compare categorical variables, while the Student’s t-test was used for normally distributed continuous variables. BMI: body mass index, HDL: high-density lipoprotein, LDL: low-density lipoprotein, eGFR: estimated glomerular filtration ratio, HOMA-IR: homeostatic model assessment for insulin resistance, SD: standard deviation, FBG: fasting blood glucose, SBP: systolic blood pressure, DBP: diastolic blood pressure, TG: triglycerides, IRI: immunoreactive insulin, SUA: serum uric acid, CVD: cardiovascular disease

-	BMI < 22.0 kg/m^2^	BMI ≥ 22.0 kg/m^2^	
Characteristic N = 2,040	N = 723	N = 1,317	p-value*
Male gender, n (%)	286 (39.6%)	595 (45.2)	0.015
Age (years)	62 ± 13	63 ± 12	0.333
BMI (kg/m^2^)	20.1 ± 1.4	25.0 ± 2.4	< 0.001
Smoking status (never/past/light/heavy), %	73.2/18.0/3.3/5.5	72.4/18.6/4.5/4.6	0.459
Drinking habits (never/occasional/light/heavy), %	48.0/28.8/14.2/9.0	45.3/27.6/17.0/10.1	0.287
History of CVD, n (%)	53 (7.3)	103 (7.8)	0.728
Hypertension, n (%)	316 (43.7)	803 (61.0)	< 0.001
SBP (mmHg)	132 ± 22	141 ± 21	< 0.001
DBP (mmHg)	78 ± 11	83 ± 11	< 0.001
Use of antihypertensive medication, n (%)	142 (19.6)	411 (31.2)	< 0.001
Hypertriglyceridemia, n (%)	63 (8.7)	282 (21.4)	< 0.001
TG (mg/dL)	82 (62-109)	99 (74-140)	< 0.001
Hypo-HDL-cholesterolemia, n (%)	12 (1.7)	75 (5.7)	< 0.001
HDL cholesterol (mg/dL)	68 ± 16	60 ± 14	< 0.001
Hyperlipidemia, n (%)	165 (22.8)	452 (34.3)	< 0.001
LDL cholesterol (mg/dL)	112 ± 30	123 ± 30	< 0.001
Use of lipid-lowering medication, n (%)	33 (4.6)	111 (8.4)	0.001
Chronic kidney disease, n (%)	45 (6.2)	136 (10.3)	0.002
eGFR (mL/min/1.73 m^2^)	81.1 ± 16.2	78.1 ± 16.4	< 0.001
Hyperuricemia, n (%)	63 (8.7)	208 (15.8)	< 0.001
SUA (mg/dL)	4.8 ± 1.4	5.3 ± 1.4	< 0.001
Use of SUA-lowering medication, n (%)	14 (1.9)	65 (4.9)	0.001
HOMA-IR	0.90 (0.60-1.34)	1.46 (0.99-2.12)	< 0.001
IRI (μU/mL)	3.8 (2.5-5.7)	6.1 (4.2-8.8)	< 0.001
FBG (mg/dL)	93 (87-102)	96 (89-104)	< 0.001

Relationship between BMI and HOMA-IR

**Figure 2:**
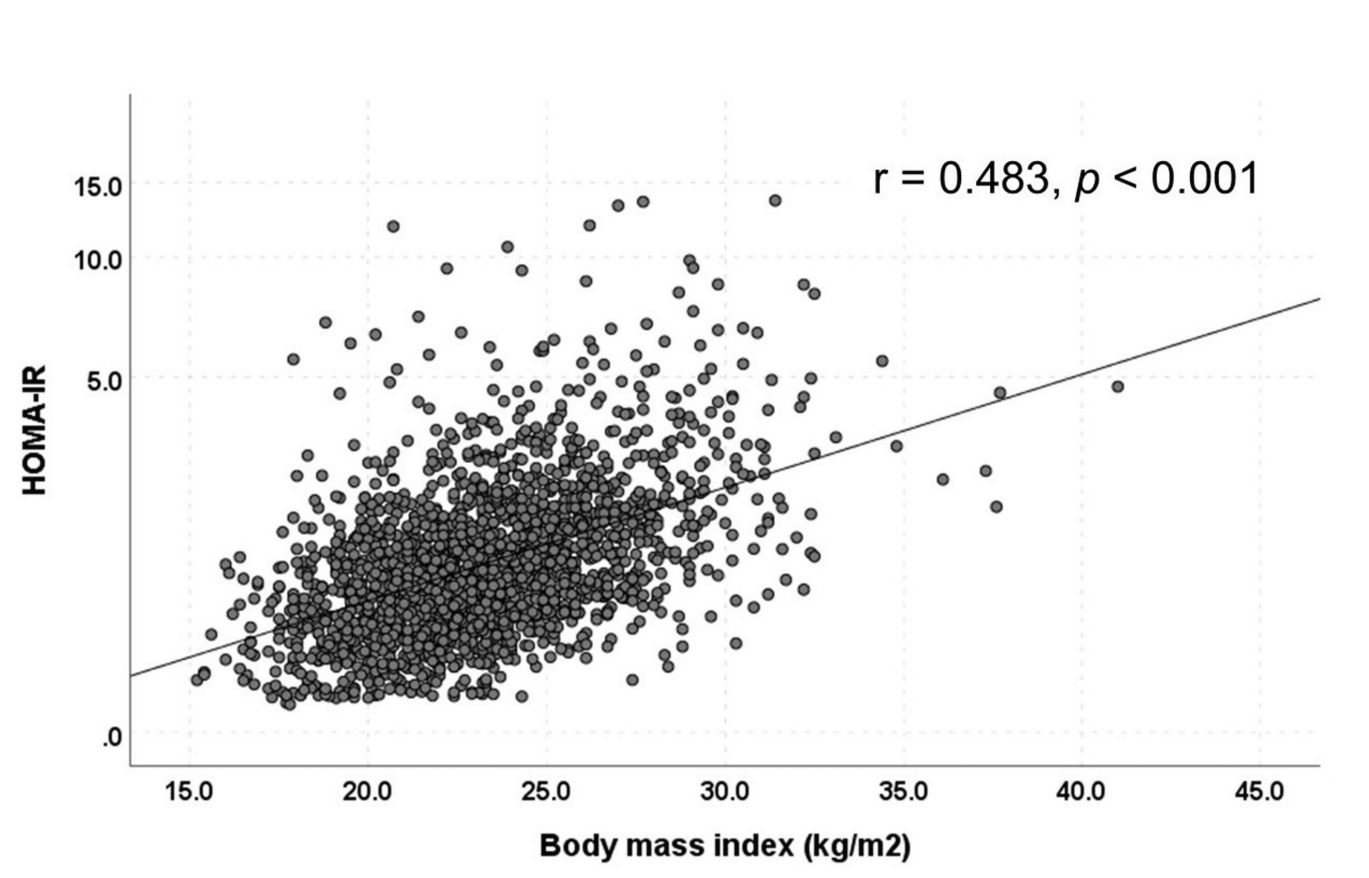
Relationship between BMI and HOMA-IR HOMA-IR increased significantly and positively with increasing BMI (r = 0.483, p < 0.001). BMI: body mass index, HOMA-IR: homeostatic model assessment for insulin resistance

Figure 2 shows the relationship between BMI and HOMA-IR. HOMA-IR increased significantly and positively with increasing BMI (r = 0.483, p < 0.001).

Association between HOMA-IR (continuous data) and all-cause mortality of subjects categorized by BMI

Table 2 shows the hazard ratios (HR) of HOMA-IR as continuous data for all-cause mortality in each group divided by a BMI of 22.0 kg/m². After adjusting for confounders including gender and age, a 1-natural log-unit increase in HOMA-IR was associated with a significantly higher HR in the group with a BMI of 22.0 kg/m² or higher (HR: 1.19; 95% confidence interval (CI): 1.02-1.40), whereas no significant association was observed in the overall cohort or the group with a BMI below 22.0 kg/m². Additionally, when adjustments were made for all potential confounders, the HR remained significantly elevated in the group with a BMI of 22.0 kg/m² or higher (HR: 1.19; 95% CI: 1.00-1.42).

**Table 2:** Association between HOMA-IR (continuous data) and all-cause mortality of subjects categorized by BMI Model 1 was non-adjusted; Model 2 was adjusted for gender and age; Model 3 was adjusted for BMI, smoking status, drinking habits, and history of CVD in addition to covariates in Model 2; and Model 4 was adjusted for hypertension, hypertriglyceridemia, hypo-HDL cholesterolemia, hyperlipidemia, diabetes, chronic kidney disease, and hyperuricemia in addition to covariates in Model 3. Data for HOMA-IR were skewed and log-transformed for analysis HOMA-IR: homeostatic model assessment for insulin resistance, BMI: body mass index, HDL: high-density lipoprotein, CVD: cardiovascular disease

-	Total	-	BMI < 22.0 kg/m^2^		BMI ≥ 22.0 kg/m^2^		-
-	N = 2,040	-	N = 723	-	N = 1,317	-	-
-	β (95% CI)	p-value	β (95% CI)	p-value	β (95% CI)	p-value	p for interaction
Events (%)	672 (32.9%)	-	245 (33.9%)	-	427 (32.4%)	-	-
Model 1	0.93 (0.83-1.04)	0.195	0.92 (0.75-1.12)	0.409	0.98 (0.84-1.14)	0.776	0.331
Model 2	1.03 (0.92-1.16)	0.587	0.89 (0.72-1.10)	0.295	1.19 (1.02-1.40)	0.027	0.001
Model 3	1.11 (0.98-1.27)	0.110	1.03 (0.83-1.28)	0.799	1.19 (1.00-1.41)	0.049	0.001
Model 4	1.10 (0.96-1.26)	0.161	0.99 (0.79-1.25)	0.945	1.19 (1.00-1.42)	0.047	0.001

An interactive relationship between BMI and HOMA-IR for all-cause mortality

Table 3 shows the HRs for death for all confounding factors, including the interaction between BMI and HOMA-IR. The interaction between HOMA-IR and BMI (HR: 1.05; 95% CI: 1.02-1.09) was significantly associated with all-cause mortality, gender, age, BMI, history of CVD, hyperuricemia, and HOMA-IR. In addition, BMI (HR: 0.94; 95% CI: 0.91-0.97) was negatively associated with mortality even after adjusting for other factors.

**Table 3:** Interactive relationship between HOMA-IR (continuous data) and BMI for all-cause mortality * Interaction between BMI and HOMA-IR. HOMA-IR was skewed and log-transformed for analysis. BMI: body mass index, HOMA-IR: homeostatic model assessment for insulin resistance, HDL: high-density lipoprotein, CVD: cardiovascular disease

-	All-cause mortality	-
Characteristic N = 2,040	β (95% CI)	p-value
Gender (male =1/female = 2)	0.71 (0.57-0.89)	0.003
Age (per 1 year)	1.12 (1.11-1.14)	< 0.001
BMI (per 1 kg/m^2^)	0.94 (0.91-0.97)	< 0.001
Smoking status (never = 0/past = 1/light = 2/heavy = 3)	1.10 (1.00-1.22)	0.061
Drinking habits (never = 0/occasional = 1/light = 2/heavy = 3)	1.02 (0.92-1.13)	0.658
History of CVD (no = 0, yes = 1)	1.48 (1.19-1.84)	< 0.001
Hypertension (no = 0, yes = 1)	1.15 (0.96-1.38)	0.122
Hypertriglyceridemia (no = 0, yes = 1)	0.89 (0.71-1.12)	0.327
Hypo-HDL-cholesterolemia (no = 0, yes = 1)	0.78 (0.54-1.14)	0.199
Hyperlipidemia (no = 0, yes = 1)	0.97 (0.81-1.15)	0.712
Chronic kidney disease (no = 0, yes = 1)	1.12 (0.89-1.39)	0.332
Hyperuricemia (no = 0, yes = 1)	1.35 (1.08-1.70)	0.010
HOMA-IR (per 1-natural-log-unit increase)	0.34 (0.16-0.71)	0.004
BMI * HOMA-IR	1.05 (1.02-1.09)	0.001

Association between HOMA-IR (categorical data) and all-cause mortality of subjects categorized by BMI

**Figure 3:**
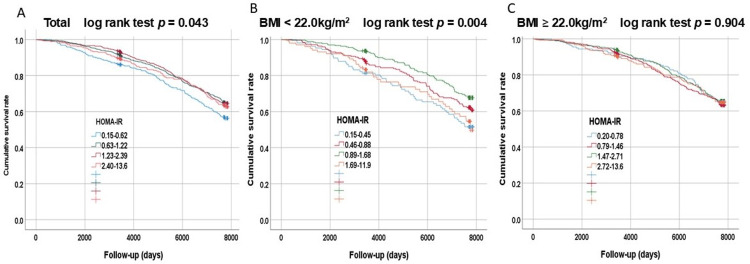
Kaplan–Meier survival curves for overall survival of subjects categorized by BMI and HOMA-IR Participants (A) were divided into two groups based on a BMI of either above (C) or below 22.0 kg/m² (B) and into four groups by one SD of HOMA-IR. BMI: body mass index, HOMA-IR: homeostatic model assessment for insulin resistance, SD: standard deviation

Figure 3 shows Kaplan-Meier survival curves for the overall survival of participants categorized by BMI and HOMA-IR. In the overall group, reduced survival was seen in the low HOMA-IR group (log-rank test p = 0.043), and in the group with BMI < 22.0 kg/m^2^, reduced survival was seen in the low and high HOMA-IR groups (log-rank test p = 0.004).

The multivariable-adjusted HRs for all-cause mortality were examined for each BMI group by dividing the HOMA-IR by one SD (Table 4). In the group with BMIs of less than 22.0 kg/m², using the third HOMA-IR as the reference, significant increases in HR (J curve) were observed in the first (HR: 1.69; 95% CI: 1.11-2.56), second (HR: 1.49; 95% CI: 1.07-2.07), and fourth HOMA-IR (HR: 2.23; 95% CI: 1.49-3.33). In the BMI of 22.0 kg/m² or higher group, using the first HOMA-IR as the reference, a significant increase in HR was observed only in the fourth HOMA-IR (HR: 1.51; 95% CI: 1.02-2.25). Again, an interaction between BMI and HOMA-IR was identified for all-cause mortality (p = 0.005).

**Table 4:** Association between HOMA-IR (categorical data) and all-cause mortality of subjects categorized by BMI HOMA-IR, four groups divided by one SD. The model was adjusted for gender, age, BMI, smoking status, drinking habits, history of CVD, hypertension, hypertriglyceridemia, low-HDL cholesterolemia, hyperlipidemia, diabetes, chronic kidney disease, and hyperuricemia. Data for HOMA-IR were skewed and log-transformed for analysis. HR: hazard ratio, CI: confidence interval, HOMA-IR: homeostatic model assessment for insulin resistance, SD: standard deviation, BMI: body mass index, HDL: high-density lipoprotein, CVD: cardiovascular disease

Total				BMI < 22.0 kg/m^2^			BMI ≥ 22.0 kg/m^2^			
-		N = 2,040	-	-	N = 723	-	-	N = 1,317	-	-
-		HR (95% CI)	p-value	-	HR (95% CI)	p-value	-	HR (95% CI)	p-value	p for interaction
Events (%)		672 (32.9%)	-	-	245 (33.9%)	-	-	427 (32.4%)	-	-
HOMA-IR		-	-	HOMA-IR	-	-	HOMA-IR	-	-	-
1^st^	0.15-0.62	1.20 (0.95-1.53)	0.122	0.15-0.45	1.69 (1.11-2.56)	0.015	0.20-0.78	Reference	-	-
2^nd^	0.63-1.22	Reference	-	0.46-0.88	1.49 (1.07-2.07)	0.020	0.79-1.46	1.18 (0.87-1.61)	0.289	0.005
3^rd^	1.23-2.39	1.06 (0.88-1.28)	0.558	0.89-1.68	Reference	-	1.47-2.71	1.22 (0.89-1.69)	0.222	
4^th^	2.40-13.6	1.35 (1.04-1.75)	0.026	1.69-11.9	2.23 (1.49-3.33)	< 0.001	2.72-13.6	1.51 (1.02-2.25)	0.042	

Association between HOMA-IR (categorical data) and all-cause mortality of subjects categorized by BMI and gender

Furthermore, as shown in Table 5, when analyzing the data separately by gender, a similar J-shaped curve pattern was observed between HOMA-IR and all-cause mortality in individuals with a BMI below 22.0 kg/m² for both men and women. On the other hand, no significant association was found in the group with a BMI of 22.0 kg/m² or higher.

**Table 5:** Association between HOMA-IR (categorical data) and all-cause mortality of subjects categorized by BMI and gender HOMA-IR, four groups divided by one SD. The model was adjusted for gender, age, BMI, smoking status, drinking habits, history of CVD, hypertension, hypertriglyceridemia, hypo-HDL cholesterolemia, hyperlipidemia, diabetes, chronic kidney disease, and hyperuricemia. Data for HOMA-IR were skewed and log-transformed for analysis. HR: hazard ratio, CI: confidence interval, SD: standard deviation, HOMA-IR: homeostatic model assessment for insulin resistance, BMI: body mass index, HDL: high-density lipoprotein, CVD: cardiovascular disease

Men N = 881							Women N = 1159					
BMI < 22.0 kg/m^2^				BMI ≥ 22.0 kg/m^2^			BMI < 22.0 kg/m^2^			BMI ≥ 22.0 kg/m^2^		
-		HR (95% CI)	-		HR (95% CI)	-	-	HR (95% CI)	-	-	HR (95% CI)	-
-		N = 286	p-value	HOMA-IR	N = 595	p-value	HOMA-IR	N = 437	p-value	HOMA-IR	N = 722	p-value
Events (%)		111 (38.8%)	-	-	227 (38.2%)	-	-	134 (30.7%)	-	-	200 (27.7)	-
1^st^	0.15-0.45	1.78 (0.96-3.33)	0.070	0.20-0.78	Reference	-	0.15-0.45	2.32 (1.23-4.37)	0.009	0.20-0.78	Reference	-
2^nd^	0.46-0.88	1.92 (1.07-3.44)	0.029	0.79-1.46	1.37 (0.94-1.99)	0.098	0.46-0.88	1.39 (0.90-2.13)	0.140	0.79-1.46	0.91 (0.52-1.58)	0.908
3^rd^	0.89-1.68	Reference	-	1.47-2.71	1.31 (0.88-1.96)	0.190	0.89-1.68	Reference	-	1.47-2.71	1.07 (0.61-1.87)	0.825
4^th ^	1.69-11.9	4.38 (1.98-9.66)	< 0.001	2.72-13.6	1.29 (0.71-2.36)	0.399	1.69-11.9	1.76 (1.09-2,85)	0.022	2.72-13.6	1.42 (0.77-2.62)	0.263

## Discussion

Our study identified a significant relationship between HOMA-IR and all-cause mortality. BMI showed a significant positive relationship with HOMA-IR while showing a negative correlation with all-cause mortality. Therefore, we examined all-cause mortality in each of the four HOMA-IR groups, divided by one SD, and further divided into two groups based on a BMI cut-off of 22.0 kg/m^2^. Notably, our analysis uncovered a distinct, nonlinear, J-shaped relationship between HOMA-IR and all-cause mortality among individuals with a BMI of less than 22.0 kg/m^2^. This complex pattern illustrated a unique trajectory where the mortality risk initially declined, reached a minimum, and then increased again, offering a detailed understanding of the intricate relationship between IR and mortality outcomes in the context of BMI. To our knowledge, this is the first study to investigate the nonlinear relationships between HOMA-IR and mortality in a non-obese population.

Not many cohort studies examine the relationship between HOMA-IR and all-cause mortality. In a cohort of 5,511 nondiabetic adults from the third U.S. National Health and Nutrition Examination Survey (NHANES) (1988-1994), followed for up to 12 years, HOMA-IR was significantly associated with all-cause mortality only in participants with a BMI below 25.2 kg/m², the median value. This association was not observed in those with a BMI of 25.2 kg/m^2^ or higher [15]. The study included 1,126 participants, with 455 deaths occurring during a median follow-up of 76 months. The analysis showed a segmented effect of HOMA-IR on all-cause mortality, with a significant inflection point at 3.59. Below this threshold, HOMA-IR was negatively associated with all-cause mortality (HR: 0.87; 95% CI: 0.78-0.97), whereas above it, HOMA-IR exhibited a positive association with all-cause mortality (HR: 1.03; 95% CI: 1.00-1.05) [20]. Among 7,085 participants aged 20 years and older from the 1999-2006 NHANES, a nonlinear J-shaped relationship between HOMA2-IR and all-cause mortality (p for nonlinearity < 0.001) was observed in the general population. In contrast, a nonlinear U-shaped relationship with all-cause mortality (p for nonlinearity < 0.001) was found in the obese population. Below the inflection point of 1.85, a negative association between HOMA2-IR and all-cause mortality was identified [21]. In our study, a J-shaped curve was identified between HOMA-IR and all-cause mortality in the group with BMIs of less than 22.0 kg/m^2^ in both genders, while a capacitive relationship was observed in the 22.0 kg/m^2^ or higher group. Variations in the number, age, and background of participants may have influenced the findings, indicating that excessively low IR could also increase the risk of mortality.

The mechanisms underlying IR in the development of all-cause mortality are believed to involve both direct atherogenic effects of insulin on the vessel walls [5], including inflammation and endothelial dysfunction [22], as well as indirect effects through factors such as obesity, blood pressure, lipids, and metabolic homeostasis [23]. IR is the core metabolic abnormality in metabolic syndrome [24], a significant risk factor for all-cause mortality. All these conditions contribute to an increased risk of premature mortality. The molecular mechanisms underlying these processes include elevated oxidative stress, altered microRNA expression, aberrant insulin signaling pathways, and mitochondrial dysfunction [25].

The variations in how HOMA-IR affects mortality across different BMI categories remain unclear. IR is closely linked to obesity metrics, including body mass. In particular, having excess body fat is recognized as a significant contributor to IR. Nevertheless, our research indicates that HOMA-IR is related to all-cause mortality even when controlling for BMI. Additionally, an unexpected and intriguing result from our study was the identification of a J-shaped relationship between HOMA-IR and all-cause mortality for individuals with a BMI of less than 22.0 kg/m^2^, with excessively reduced HOMA-IR also associated with increased mortality. In individuals without obesity, IR may be caused by factors other than BMI [21]. Notably, lower IR levels were linked to lower fasting glucose levels [26]. The rise in epinephrine due to repeated hypoglycemia induced endometrial thickening and smooth muscle cell proliferation in Goto-Kakizaki rats; however, glucose injection was able to counteract hypoglycemia and prevent endometrial thickening [27]. In addition, excessively low IR reflects underlying factors such as malnutrition, reduced muscle mass, or chronic conditions (e.g., anemia, cancer, or chronic inflammatory diseases), which induce hypoglycemia and all-cause mortality [28]. We postulate that other factors could have influenced mortality in the first quintile HOMA-IR group.

Although this study provides valuable insights into the rural population of Japan, it has several limitations. First, the sample was predominantly comprised of relatively healthy middle-aged and elderly individuals residing in rural areas of Japan who took part in health checkups. Consequently, it may not accurately represent the general population. Second, a cross-sectional design was employed to assess baseline characteristics and HOMA-IR levels at the initial visit. However, HOMA-IR levels and some covariates may vary over time and could have changed during the extended follow-up period. Additionally, HOMA-IR is not as accurate as the euglycemic clamp method, which is the gold standard for diagnosing IR [29]. Although it is difficult to use in large-scale clinical studies due to its limitations. This might lead to underestimating the study's significance due to nondiscriminatory misclassification bias. Thirdly, the study assumed that all deaths were recorded in Japan's Basic Resident Register, regardless of cause. This implies that individuals who emigrated during the study period might have been excluded from the analysis. Furthermore, the baseline assessment incorporated various potential confounders, such as medications, pre-existing conditions, and lifestyle factors, all previously linked to mortality. While efforts were made to control for these confounders through baseline physical examinations, there may still be unmeasured factors that were not captured in this study. Consequently, further research is necessary to investigate the impact of these unexamined variables (e.g., causes of mortality). Finally, due to the relatively small sample size and limited number of mortality cases, this study may have underestimated the potential causal relationship between HOMA-IR levels and all-cause mortality.

## Conclusions

HOMA-IR stratification showed a correlation with all-cause mortality. Very low levels of IR were linked to higher mortality rates in people with BMIs of less than 22.0 kg/m^2^. It is important to emphasize that keeping the levels of IR above an excessively low threshold is important for the health of these populations.
